# The Treatment of Disease by Animal Extracts

**Published:** 1894-02-17

**Authors:** 


### CURRENT MEDICAL TOPICS.

### THE TREATMENT OF DISEASE BY ANIMAL EXTRACTS.

THE paper read by Dr. Abraham on the use of thyroid gland in the treatment of diseases of the skin at the Medical Society considerably enlarged the published experience in regard to this branch of therapeutics. What appeared quite clear, both from the paper itself and from the discussion which followed, was that in regard to none of the skin diseases, which had been made the subject of experiment, had thyroid gland any specific action comparable to that which it possesses over myxœdema. That the thyroid gland contains a substance which is capable of profoundly modifying certain states of innutrition is one of those curious facts which, however much we may be in the dark as to its causation or *modus operandi*, must be accepted as definitely proved. No one can watch the rapid and striking change which takes place in a typical case of myxœdema treated by thyroid gland, and observe how, after a relapse, this improvement can be reproduced time after time, on renewal of the treatment, without acknowledging that there is something in the gland which has an exceedingly active influence in restoring patients suffering from the disease to a condition of apparent health. It must, however, be borne in mind that, both practically and theoretically, the use of this substance as a specific in myxœdema stands on an entirely different footing from its use in other conditions. As regards myxœdema there is a certain *a priori* probability of its being of service. No one knows what is the function of the thyroid, but it is a ductless gland, and whatever it produces must flow back again in some form or other into the circulation, so that although it was a matter of some surprise to find that the removal of so apparently unimportant a body, so functionless a gland, if one may use the term, should produce such disastrous effects upon the economy; yet when these effects were once recognised it was an easy inference, speaking of course after the event, to see that the restoration to the body of the secretion of the gland might probably undo the mischief; and, when success followed the operation of ingrafting, the extension of the idea to the seemingly analogous case of myxœdema was but a final step in a logical series of inductions. The case, however, is quite otherwise in regard to many of the multifarious therapeutic uses which have been proposed for thyroid gland since its utility was established in that disease; for although there is a certain reasonableness in the considerations which lead to its employment in some conditions, it is to be feared that in regard to many its prescription has been more like a bow drawn at a venture.

There were, however, a fair number of cases of skin diseases, especially of the dry and chronic type, in which definite improvement was observed, and several cases of psoriasis were rapidly cured; in others, however, the amelioration which took place seemed to occur no more quickly than under other forms of treatment, and in some no effect was produced. Among the cases in which Dr. Abraham observed at any rate temporary improvement were some of leprosy.

Considering the influence of the drug, for so it must now be called, in softening the skin and modifying its nutrition in cases of myxœdema, an influence which sometimes goes so far as to cause extensive desquamation, it is not to be wondered at that dry and scaly psoriasis was one of the first skin affections in which thyroid feeding was made use of; and even now, among all the heterogeneous diseases for which it has been tried, psoriasis remains the one in which, always excepting myxœdema, relief has been most frequently observed, although that may in part be due to its being the one in which it has been most frequently prescribed.

It seems pretty clear from the whole tone of the discussion that it is impossible to say beforehand in which cases benefit is likely to result. It was mentioned, however, that the completely chronic ones—those which seemed to have tried everything—were most under its influence, and that the feebler patients, those whose general health was distinctly below the mark, were more often helped than stronger folk; also that its influence for good was more marked among children than older people. None of these conclusions, however, were borne out by the cases observed by Dr. Abraham.

There was a general consensus of opinion that the remedy was one of considerable potency, and not to be trifled with; and this warning is perhaps all the more necessary at the present time, now that it can be so readily obtained by anyone in the form of tabellæ, and administered just in the same way as any other drug. Under the influence of an over-dose nausea and pyrexia tend to be induced; under any circumstances it causes considerable exhaustion. Tremors are sometimes produced, and its effect upon the heart must not be forgotten.

Ever since the publication of Brown-Sequard's communication on the effects produced in man by subcutaneous injections of a liquid obtained from the testicles of animals, there has been a growing tendency to experiment on the therapeutic uses of various animal juices, and many of these extracts are now to be had as ordinary commercial products. It must be confessed, however, that there is a great deal which is purely speculative, and open to much suspicion, in such proceedings as the administration of spermin for senile debility, cerebrine for nerve diseases, cardin for cardiac weakness, &c.. In many cases, at any rate, suggestion seems to have played a not inconsiderable part, and in all we are absolutely without clue, except so far as a vague analogy may guide us, as to what effect to look for, or in what sort of cases to expect benefit. Thyroid medication, however, is different. In myxœdema it is a certainty; in psoriasis and other chronic nutritional disorders of the skin it is based on our experience of its profound influence over the nutrition of the skin as observed during its use in myxœdema; in certain conditions of the brain also its utility is made probable by the well-known brightening of the intellectual faculties which follows its use in the same disease; but in the whole range of other diseases in which it has been tried it is to be feared that thyroid stands much on the same footing as other animal extracts, as a thing which may or may not be useful, but in regard to which it is impossible to say beforehand in what case it will do good.

